# The Crosstalk between the EGFR and IFN-γ Pathways and Synergistic Roles in Survival Prediction and Immune Escape in Gliomas

**DOI:** 10.3390/brainsci13091349

**Published:** 2023-09-20

**Authors:** Xingang Zhou, Tingyu Liang, Yulu Ge, Yu Wang, Wenbin Ma

**Affiliations:** 1 Department of Pathology, Beijing Ditan Hospital, Capital Medical University, Beijing 100015, China; zhouxg1980@126.com; 2 Department of Neurosurgery, Center for Malignant Brain Tumors, National Glioma MDT Alliance, Peking Union Medical College Hospital, Chinese Academy of Medical Sciences and Peking Union Medical College, Beijing 100730, China; lzn13391610953@126.com (T.L.); ywang@pumch.cn (Y.W.); 3 Eight-Year Medical Doctor Program, Chinese Academy of Medical Sciences and Peking Union Medical College, Beijing 100730, China; ge-yl19@mails.tsinghua.edu.cn

**Keywords:** glioma, EGFR, IFN-γ, tumor microenvironment, classification

## Abstract

Glioma is the most common primary malignant brain tumor. The poor prognosis of gliomas, especially glioblastoma (GBM), is associated with their unique molecular landscape and tumor microenvironment (TME) features. The epidermal growth factor receptor (EGFR) gene is one of the frequently altered loci in gliomas, leading to the activation of the EGFR signaling pathway and thus, promoting the genesis of gliomas. Whether there exist factors within the TME that can lead to EGFR activation in the context of gliomas is currently unexplored. In total, 702 samples from The Cancer Genome Atlas (TCGA) and 325 samples from The Chinese Glioma Genome Atlas (CGGA) were enrolled in this study. Gene signatures related to EGFR signaling and interferon-γ (IFN-γ) response were established via the LASSO-COX algorithm. Gene Set Enrichment Analysis (GSEA) and Gene Ontology (GO) analysis were applied for function exploration. Kaplan–Meier (KM) curves and single sample GSEA (ssGSEA) of immune cell subpopulations were performed to analyze the prognosis and TME characteristics of different subgroups. Moreover, Western blotting (WB) and flow cytometry (FCM) demonstrated the correlation between IFN-γ and EGFR signaling activation and the subsequent induction of programmed death ligand 1 (PD-L1) expression. An EGFR signaling-related risk score was established, and a higher score was correlated with poorer prognosis and a more malignant phenotype in gliomas. Biological function analysis revealed that a higher EGFR-related score was significantly associated with various cytokine response pathways, especially IFN-γ. Long-term (7 days) exposure to IFN-γ (400 ng/mL) induced the activation of EGFR signaling in the u87 cell line. Next, an IFN-γ response-related risk score was established; the combination of these two scores could be used to further reclassify gliomas into subtypes with different clinical features and TME features. Double high-risk samples tended to have a poorer prognosis and more immunosuppressive TME. Additionally, FCM discovered that the activation of EGFR signaling via EGF (100 ng/mL) could trigger PD-L1 protein expression. This research indicates that IFN-γ, an inflammatory cytokine, can activate the EGFR pathway. The combination of EGFR signaling and IFN-γ response pathway can establish a more precise classification of gliomas.

## 1. Introduction

Glioma is the most common primary malignant brain tumor, accounting for approximately 80–85% of malignant brain tumors in adults [1]. It possesses high heterogeneity and consists of multiple subtypes of tumor cells. Each cell subtype is genetically and functionally different with a unique immunological landscape, such as differences in microglia or macrophage composition and T cell infiltration [2,3]. Its striking cellular heterogeneity, combined with its aggressive nature, contributes broadly to the failure of immunotherapy and molecular targeted therapies. According to the fifth edition of the World Health Organization classification of tumors of the central nervous system (WHO CNS5) [4], the median survival time of glioblastoma (GBM) did not exceed 15 months, with surgical resection, radiotherapy, and chemotherapy [5].

Epidermal growth factor receptor (EGFR) is a transmembrane tyrosine kinase located on the chromosome band 7p12. Amplification of EGFR has been observed in approximately 34–39% of GBMs [6,7,8,9], which always leads to the overexpression of EGFR and subsequent activation of downstream signaling pathways. Given the important role of EGFR amplification in glioma progression, EGFR amplification has been incorporated as one of the criteria for molecular classification of GBM in the WHO CNS5 classification [4]. Activation and autophosphorylation of EGFR result in the recruitment of downstream pathway proteins [10]. The downstream pathways of EGFR signaling not only contribute to DNA synthesis and cell proliferation [11] but also exert an influence on the tumor microenvironment (TME). For instance, EGFR signaling has been implicated in promoting macrophage infiltration within the tumor, via the chemokine ligand 2 (CCL2) [12]. Moreover, studies have shown that the activation of EGFR induces the secretion of programmed death ligand 1 (PD-L1) to inhibit the function of T cells [13]. In summary, studies reveal that EGFR signaling can reshape the TME [14], while the role of TMEs and cytokines in the activation of EGFR signaling remain unclear.

Interferon-γ (IFN-γ) is an important component within the TME that exhibits a dual role in glioma progression. First, this cytokine demonstrates the ability to directly inhibit the proliferation and invasion of glioma cells [15,16]. Second, IFN-γ in the TME is necessary for immune cells to maintain their tumor-killing activity. Studies have shown that the absence of IFN-γ in chimeric antigen receptor (CAR) T cells may hamper the in vivo antitumor activity and the activation of host immune cells [17]. Additionally, the presence of the IFN-γ receptor signaling pathway within GBMs is essential for CAR T therapy [18]. Furthermore, compared to adjuvant therapy alone, neoadjuvant programmed cell death protein 1 (PD-1) blockade is associated with the upregulation of IFN-γ-related gene expression to elevate the antitumor activity [19]. Thus, IFN-γ is an important cytokine induced by immunotherapy. However, various immunotherapies have failed to improve glioma clinical outcomes. It is noteworthy that IFN-γ also serves as a significant inducer of PD-L1 expression in the TME. The increased PD-L1 expression, in turn, leads to T cell dysfunction and apoptosis, thus contributing to the suppression of inflammatory responses and facilitating tumor immune evasion [13,20].

Studies have reported some communication between the IFN-γ and EGFR signaling pathways. In A431 cells, an epidermoid carcinoma cell line, IFN-γ induced a rapid and reversible tyrosine phosphorylation of EGFR [21]. In ovarian cancer cell lines, although IFN-γ reduced cell proliferation by 30–40%, it strikingly increased the EGFR expression, including cell surface receptors and total cellular receptors [22]. However, in a breast cancer cell line MDA468, IFN-γ inhibited cell proliferation while reducing the number of available EGFR binding sites, without any change in the EGFR affinity [23]. As for glioma, no studies have reported that IFN-γ stimulation could directly modulate EGFR expression or EGFR activity. Since IFN-γ can both inhibit the malignant phenotype of glioma cells and maintain the killing activity of immune cells, and EGFR serves as an important factor in glioma progression, it is necessary to explore the effect of IFN-γ on EGFR in gliomas. The relationship between these two factors is instructive for glioma treatment. For example, if IFN-γ can increase EGFR activity, IFN-γ may upregulate PD-L1 expression by elevating the activity of the EGFR pathway. Therefore, inhibition of the EGFR pathway may somewhat reduce PD-L1 expression caused by IFN-γ stimulation, thus enhancing the tumor-killing activity of infiltrated immune cells.

This research aimed to explore the relationship between IFN-gamma and EGFR in the context of gliomas, followed by an investigation of their effects on the TME. The analysis of RNA sequencing data sourced from the Cancer Genome Atlas (TCGA) and The Chinese Glioma Genome Atlas (CGGA) databases revealed a noteworthy correlation between the activation of the EGFR pathway and IFN-γ pathway, and the activation of these two pathways significantly affected the immune infiltration and the immune checkpoint gene expression within the TME. To validate this finding, experiments were conducted using the U87 cell line, followed by validating the results that IFN-γ can activate the EGFR pathway and activation of the EGFR pathway can upregulate PD-L1 expression. The results further support the notion that inflammatory molecules within TME could potentially influence the specific molecular mechanisms underlying tumor progression. Through our current research, we provide the promising possibility of a combination of immunotherapy and EGFR-targeted medicine in gliomas.

## 2. Methods

### 2.1. Samples and Datasets

RNA sequencing data and corresponding clinical information of 702 glioma samples from TCGA-GBM and TCGA-LGG (https://portal.gdc.cancer.gov/, accessed on 1 May 2023) and 325 glioma samples from CGGA-325 (https://www.cgga.org.cn, accessed on 1 May 2023) were retrospectively enrolled in this study. TCGA mainly covers European and American races. Therefore, we selected CGGA, the largest glioma database in China, to validate the results obtained from TCGA. All samples with RNA sequencing data and clinical data were enrolled without selection. Clinical information included age, gender, histology, WHO grade, overall survival, chromosome 1p19q codeletion status, and isocitrate dehydrogenase (IDH) mutation status. Overall survival was estimated from the date of diagnosis to the date of last follow-up or the date of death. These two cohorts were independent with no patients overlapping, and were widely used in glioma research due to comprehensive data. In total, 186 EGFR signaling pathway-related genes were obtained from the Genecards Database (https://www.genecards.org/, accessed on 1 May 2023) [24].

This study was conducted based on data from publicly available database sources and based on experiments using cell lines. Ethical approval was therefore not required.

### 2.2. Construction of EGFR-Related and IFN-γ-Related Prognostic Gene Signatures

In total, 186 EGFR signaling pathway-related genes were obtained from the Genecards Database. In all the following processes, genes were selected based on statistical significance, namely *p*-value less than 0.05. To analyze whether expression, a continuous variable, had an effect on survival, univariate Cox regression analysis was performed in the TCGA and CGGA cohorts separately. Genes with significantly different expression patterns based on EGFR amplification status in IDH1-wildtype GBMs were screened out. In total, 23 genes that were retained in the results of all the above processes were selected.

A risk model was established using the TCGA dataset, after which the parameters obtained from this risk model were substituted into the CGGA dataset for validation. To prevent overfitting in the generation of a risk model with too many variables, the least absolute shrinkage and selection operator (LASSO) machine learning algorithm [25] was employed to dimensionally downscale the 23 genes. To find independent prognostic factors in the presence of multiple variables, multivariate Cox regression analysis was used for the remaining 9 genes. Finally, 4 genes that independently affected prognosis were retained and used to construct a risk model. The risk score for all patients was determined by summing the regression coefficients of the selected genes multiplied by the corresponding expression values.

For the IFN-γ-related risk score, the process was similar to that just used for EGFR. In total, 200 IFN-γ response genes were involved, followed by selection via multivariate Cox regression analysis, LASSO, and univariate Cox regression analysis to construct IFN-γ-related prognostic gene signatures. In summary, 4 genes and 8 genes were used to establish EGFR-related and IFN-γ-related prognostic gene signatures, respectively.

### 2.3. Biological Function and Signaling Pathway Analysis

Patients from both datasets were divided into high-risk and low-risk groups based on the median risk score as a threshold. Pearson correlation analysis was conducted to determine genes that were positively correlated with the risk score (*R*  >  0.4, *p*  <  0.05). The two variables were considered to be relatively strongly correlated when the R-value was greater than 0.4, and genes selected for the ensuing pathway enrichment could not be too few, with 0.4 used as the cut-off criterion. The TCGA and CGGA cohorts were analyzed separately. The correlation results were used individually for Gene Set Enrichment Analysis (GSEA). The intersection of positively correlated gene sets from the two cohorts was taken for gene ontology (GO) analysis. This was due to the different data entry requirements of GSEA and GO, where GSEA requires a gene set with correlation coefficients, while GO only requires a gene set containing the gene name. The database of all pathway enrichments came from the MSigDB database version 7.2. The pathway enrichment was conducted using the ClusterProfiler R package [26]. This package is conducive to enrichment analysis based on a given gene set. PROGENy was used to calculate the pathways activity score (N = 11) [27]. The PROGENy algorithm can infer the activation of 11 tumorigenesis-related signaling pathways based on gene expression.

### 2.4. Comprehensive Analysis of Immune and Molecular Characteristics

The ssGSEA method was used for immune cell infiltration and inflammation activity [28]. Also, the ESTIMATE R package was used to evaluate the purity of gliomas and the proportion of infiltrating immune cells and stromal cells [29].

### 2.5. Cell Culture for Western Blot (WB) and Flow Cytometry (FCM)

The GBM cell line u87 was collected from the Institute of Biochemistry and Cell Biology, Chinese Academy of Science, and was cultured in Dulbecco’s modified Eagle’s medium (DMEM, Corning, NY, USA) supplemented with 10% fetal bovine serum (FBS, Gibco, Grand Island, NY, USA) and 1% penicillin–streptomycin (Gibco). To analyze the function of IFN-γ on EGFR signaling activation, the u87 cell was exposed to IFN-γ (400 ng/mL, PeproTech, Cranbury, NJ, USA) for 7 days. Next, total protein was extracted for WB assay, and whole-cell lysates were prepared on ice in RIPA buffer. A microplate spectrophotometer (Infinite M200 PRO, Tecan, Männedorf, Switzerland) was used to determine the protein concentration via Coomassie Brilliant Blue. An amount of 40 mg of total protein from cell lysates was loaded on a 10% SDS-PAGE gel and then transferred to the PVDF membrane (Merck Millipore, Burlington, MA, USA). After 5% skimmed milk closure, the primary antibody was diluted with 1X TBST (Tris-buffered saline with Tween-20). Primary antibodies included EGFR (Abcam ab52894, 1:1000), p-EGFR (Abcam ab32430, 1:1000), and glyceraldehyde 3-phosphate dehydrogenase (GAPDH) (Proteintech 60004-1-Ig, 1:10,000). The membrane was incubated with primary antibodies at 4 °C overnight and then with horseradish peroxidase-conjugated secondary antibodies (ZSGB-BIO, Beijing, China) for 1 h at room temperature. The ECL Western Blotting Detection System (Bio-Rad, Hercules, CA, USA) was used to visualize protein signals. GAPDH was used as the loading control to quantify relative protein levels. Additionally, for flow cytometry (FCM) analysis, after exposure to Epidermal Growth Factor (EGF) (100 ng/mL for 48 h), 1 × 10^5^ u87 cells in EGF treatment and negative control (NC) groups were harvested and washed twice before PD-L1 antibody (BioLegend, PE anti-human PD-L1, No. 393608) staining for 30 min. Unbound antibodies were then washed out by PBS, and PD-L1 protein expression on the tumor cell surface was tested by FCM.

### 2.6. Statistical Analysis

All statistical analyses were performed using R software (version 4.1.1; https://www.r-project.org/, accessed on 1 May 2023), a free software environment for statistical calculation and graphics. The log-rank test and Kaplan–Meier method were used to evaluate survival time and calculate survival differences, which were conducted via R package survival [30]. The correlation between risk score and gene expression was tested using Pearson correlation analysis. The continuous variables between the two groups were compared using Student’s *t*-test. For categorical variables, Fisher’s exact and Chi-square tests were used for group comparison. A *p*-value of less than 0.05 was considered statistically significant.

## 3. Results

### 3.1. Establishment of an EGFR Pathway-Related Prognostic Gene Signature

**Figure 1:**
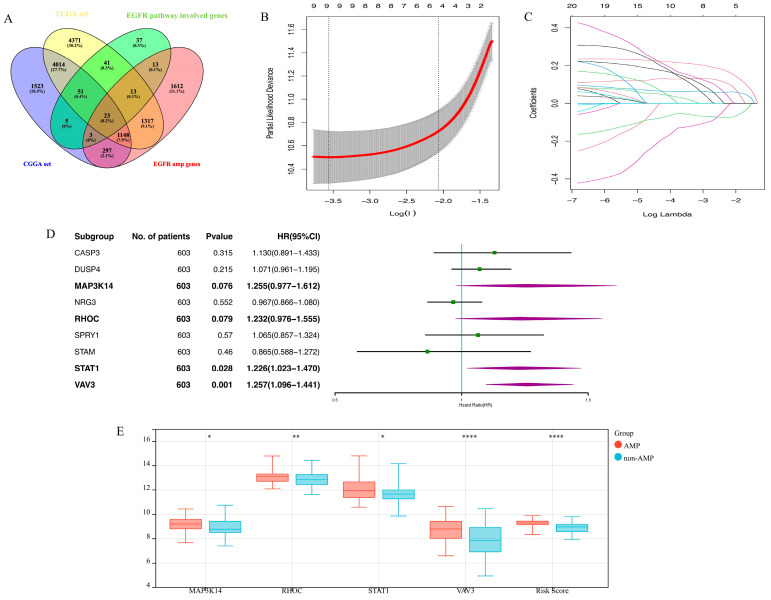
The establishment of EGFR pathway-related prognostic gene signature. (**A**). The screening process of the 23 EGFR pathway-related prognostic genes; (**B**,**C**). LASSO coefficient profiles of the remaining 23 genes; (**D**). Multivariate Cox regression analysis of the remaining 9 genes in the TCGA cohort; (**E**). The 4 left genes and the risk score distribution in the EGFR amplification and non-amplification groups (* means *p* < 0.05; ** means *p* < 0.01; **** means *p* < 0.0001).

A total of 186 genes involved in the EGFR signaling pathway were included based on information obtained from the Genecards Database. First, 74 prognosis-related genes were identified using the univariate Cox regression. Among these genes, 23 genes that showed significant differences in expression based on the EGFR gene amplification status were sorted out (Figure 1A). In total, 4 final candidate genes, MAP3K14, RHOC, STAT1, and VAV3 (Figure 1B–D) were determined using the LASSO algorithm and subsequent multivariate Cox regression analysis. The risk score was constructed using these genes and their corresponding Cox regression coefficients via the formula: 0.23 × MAP3K14^expr^ + 0.21 × RHOC^expr^ + 0.20 × STAT1^expr^ + 0.23 × VAV3^expr^. Moreover, there was a significant difference in the expression of four candidate genes and the risk score between the EGFR amplified group and the non-amplified group (*p* < 0.05, Figure 1E). The overall process of constructing EGFR-related signatures is demonstrated in Supplementary Figure S1.

### 3.2. Clinic Pathological Features Related to the EGFR Pathway-Related Prognostic Gene Signature in Gliomas

**Figure 2:**
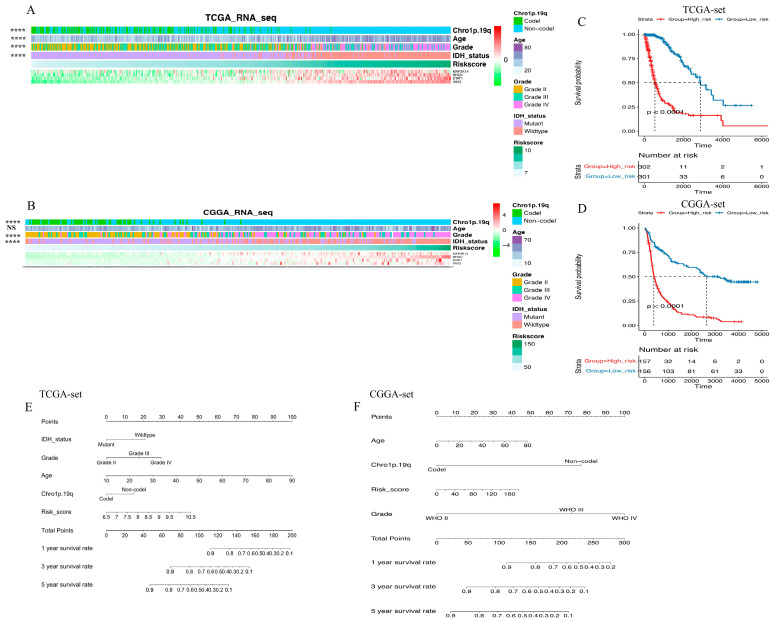
The risk score distribution and prognosis prediction of EGFR pathway-related risk score. (**A**,**B**). The expression pattern of risk score and 4 genes in glioma samples in the TCGA set (**A**) and the CGGA set (**B**); (**C**,**D**). Kaplan–Meier curves present risk score is negatively related with prognosis in the TCGA set (**C**) and the CGGA set (**D**); (**E**,**F**). The model, including risk score, demonstrates that the nomogram possesses superior predictive ability and can facilitate clinical decision-making in the TCGA set (**E**) and the CGGA set (**F**) (**** means *p* < 0.0001, NS means no significance).

To investigate the clinical and pathological relevance of the gene signature, the correlation between risk score and various clinicopathological factors was evaluated in this study. Figure 2A,B illustrates the ordering of patients based on the risk score in both datasets. The results revealed that risk score was positively associated with age at diagnosis, and lower risk score was significantly associated with low-grade gliomas, IDH mutation, and chro 1p/19q codeletion. This suggests that the EGFR pathway-based signature predicts the malignant phenotype. For further evaluation of the correlation between this gene signature and patient survival time, all glioma patients were classified into high-risk or low-risk groups according to the median cut-off point. Compared to the high-risk group in the TCGA set, the low-risk group had a significantly better prognosis (log-rank *p* < 0.0001, Figure 2C). To enhance the practicality of clinical application for individual glioma patients, a nomogram model was developed, which incorporated prognostic factors including risk score to predict overall survival at 1, 3, and 5 years (Figure 2E). The model demonstrated that the nomogram had superior predictive ability and could facilitate clinical decision-making. In addition, the above conclusions were validated in the CGGA independent cohort (Figure 2D,F).

### 3.3. Biological Processes and Signaling Pathway Analysis

**Figure 3:**
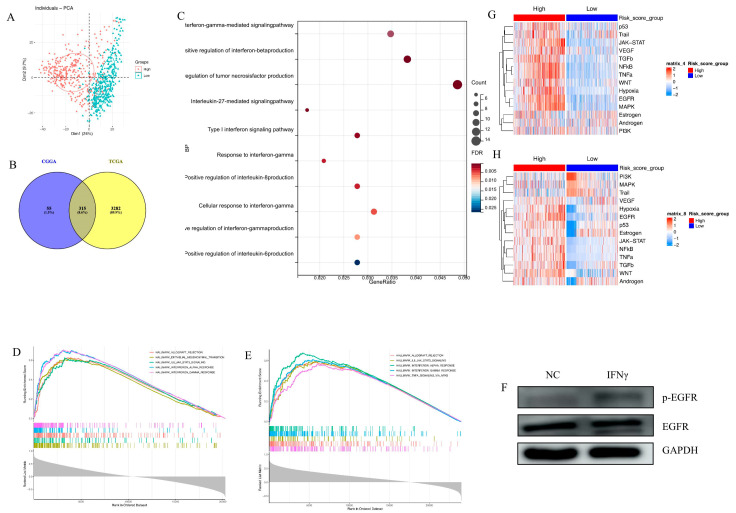
The biological function of EGFR pathway-related prognostic gene signature. (**A**). There is separation between the high-risk and low-risk groups in the TCGA cohort; (**B**). The 315 overlapped genes positively associated with risk score (*R* > 0.4, *p* < 0.0001) between the TCGA and CGGA sets; (**C**). BP analysis indicates that risk score is positively related with multiple cytokines pathways; (**D**,**E**). GSEA reveals the relationship between risk score and IFN-γ response pathway in the TCGA set (**D**) and the CGGA set (**E**); (**F**). WB assay demonstrates that long-term exposure to IFN-γ (400 ng/mL, 7 days) can trigger the activation of EGFR pathway in u87 GBM cell line; (**G**,**H**). Heatmap of signaling pathway activity scores by PROGENy in the TCGA set (**G**) and the CGGA set (**H**).

Principal component analysis (PCA) of the transcriptome demonstrated that high-risk and low-risk groups had different transcriptomic expression profiles in the TCGA cohort (Figure 3A). The CGGA cohort had a similar PCA result (Figure not shown). Given that the transcriptome tumor underlies biologic characteristics, for example, higher oncogene transcription leads to a more malignant phenotype, the PCA result suggests that high-risk and low-risk groups may have different biological profiles.

To further explore the distinct biology, Pearson correlation analysis between risk score and other genes in whole genome gene profiling was conducted. In total, 315 positively correlated genes (*R* > 0.4, *p* < 0.05), intersecting with the TCGA and CGGA populations, were selected for GO analysis (Figure 3B). Biological process (BP) analysis pointed out that multiple cytokine-related pathways were positively correlated with the EGFR pathway-related risk score, especially IFN-γ-related signaling (Figure 3C). GSEA analysis in the TCGA and CGGA cohorts showed that the gene signature was enriched in IFN-γ response signaling in both cohorts (Figure 3D,E). The long-term exposure to IFN-γ (400 ng/mL, 7 days) triggered the activation of the EGFR pathway in the u87 GBM cell line (Figure 3F), which provided further evidence for the findings obtained by pathway enrichment.

Finally, NF-κB, JAK-STAT, and TNFα signaling pathways were significantly activated in the high-risk group (Figure 3G,H). All the results revealed that the EGFR pathway-related prognostic gene signature was linked with various malignant pathways and tumor malignant behaviors.

### 3.4. Establishment of an IFN-γ-Related Prognostic Gene Signature

**Figure 4:**
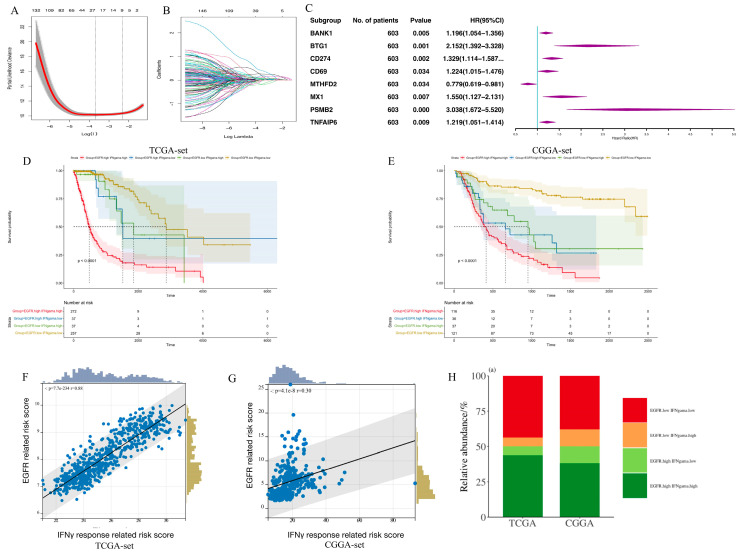
The establishment of IFN-γ response pathway signature and the relationship between EGFR pathway-related and IFN-γ response pathway prognostic gene signature. (**A**,**B**). LASSO coefficient profiles of IFN-γ response genes; (**C**). Multivariate Cox regression analysis of the selected genes in the TCGA set; (**D**,**E**). The combination of EGFR pathway-related and IFN-γ response pathway signatures can divide glioma samples into 4 groups with distinct prognosis in the TCGA set (**D**) and the CGGA set (**E**); (**F**,**G**). The relationship between the above two signatures in the TCGA set (**F**) and the CGGA set (**G**); (**H**). The majority of patients exhibit either high or low scores in both signatures, with only a small proportion of patients displaying a combination of one high and one low score in the TCGA and CGGA sets.

Based on the results of the pathway analysis, the combination between the EGFR-related signature and the IFN-γ response pathway was investigated. The process of setting the EGFR-related risk score was repeated, but based on 200 IFN-γ response genes (Figure 4A–C). In total, eight genes were left to construct an IFN-γ-related prognostic gene signature. The overall process of constructing IFN-γ-related signatures is demonstrated in Supplementary Figure S2. Next, the combined function of the above two signatures was explored. Prognostic analysis subsequently revealed distinct survival outcomes among these patient groups. The patients with low scores exhibited the most favorable prognosis, those with high and low scores displayed intermediate prognosis, and those with high scores demonstrated the poorest prognosis in both the TCGA and CGGA RNA-seq sets (Figure 4D,E, *p* < 0.0001). This indicated that the combination of these two signatures had a strong prognostic predictive power. Notably, a strong positive correlation was observed between the EGFR-related signature and the IFN-γ-related signature (Figure 4F,G; TCGA: *R* = 0.88, *p* < 0.0001; CGGA: *R* = 0.30, *p* < 0.0001). To further explore this relationship, glioma patients were stratified based on the median values of both scores. The majority of the patients exhibited either high or low scores in both signatures, and only a small proportion of patients displayed a combination of high and low scores (Figure 4H).

### 3.5. Immune Cell Infiltration and Inflammatory Profiles Related to the Gene Signature

**Figure 5:**
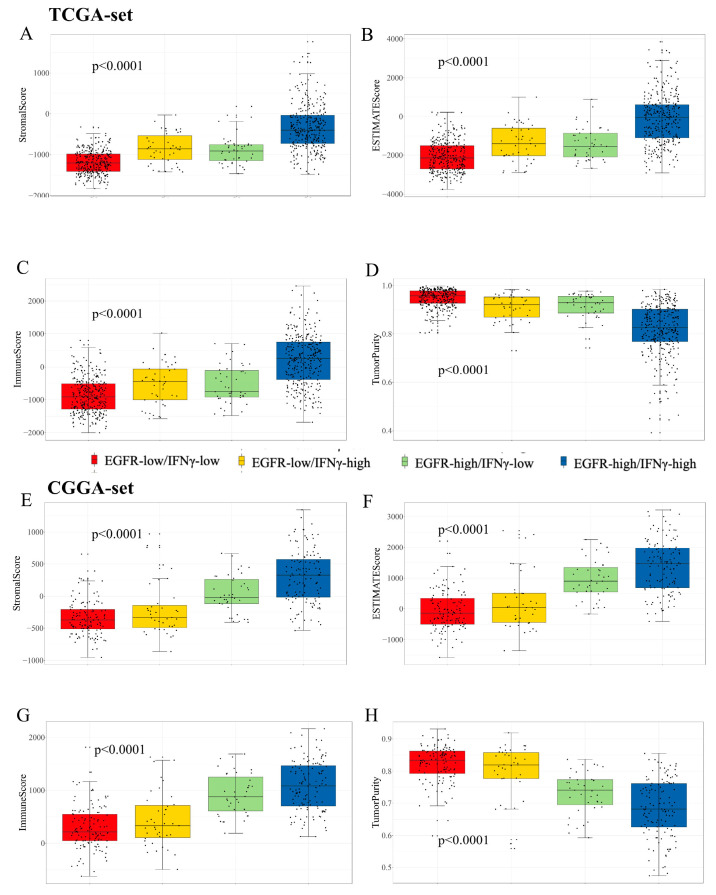
Differences among immune phenotypes of combination of the two signatures in terms of four glioma immune microenvironment signatures. (**A**–**D**). The comparison of the ESTIMATE, immune and stromal scores and tumor purity among distinct groups in the TCGA cohort; (**E**–**H**). The validation of above results in the CGGA cohort.

**Figure 6:**
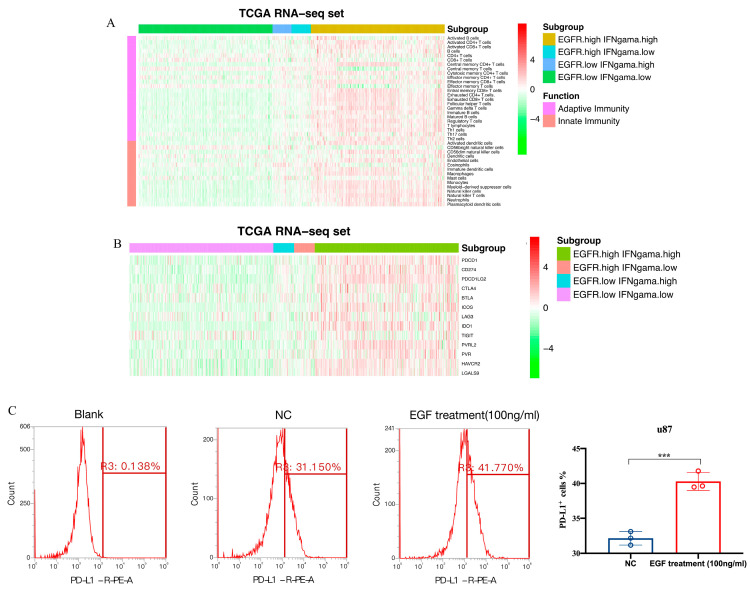
Immune cell infiltration and inflammatory profiles related to the gene signatures. (**A**). The distribution of various immune cell subpopulations in distinct groups; (**B**). The distribution of immune checkpoint molecules in different groups; (**C**). The flow cytometry verifies the activation of EGFR pathway via EGF (100 ng/mL 48 h) can upregulate PD-L1 expression on the surface of u87 cell line (*** means *p* < 0.001).

The TME characteristics of gliomas were assessed using the ESTIMATE algorithm [29]. The results showed that in the TCGA dataset, a higher score was observed in the patients with high scores (Figure 5A), while immune and stromal scores were also higher in the patients with high scores (Figure 5B,C). The patients with low scores had higher tumor purity (Figure 5D), which predicted a better prognosis [31]. Similar results were found in the CGGA dataset (Figure 5E–H). For the distribution of immune cell subsets, various immune cells were enriched in the high-risk group, such as memory CD4^+^ and CD8^+^ T cells (Figure 6A). However, exhausted T cells and regulatory T cells were also enriched in the high-risk group. Additionally, high-risk patients expressed higher levels of immune checkpoint molecules (Figure 6B), such as PD-1, PD-L1, and IDO1, which further suggests stronger immunosuppression in the high-risk group. To further validate the results, flow cytometry (FCM) was used to analyze the protein expression of PD-L1 on the surface of the u87 cell line. The result revealed that EGF (100 ng/mL, 48 h), an agonist of EGFR signaling, could remarkably upregulate PD-L1 protein expression (Figure 6C).

## 4. Discussion

Recently, the discovery of the lymphatic system in the central nervous system (CNS) has challenged the notion that CNS is an immune-privileged site [32,33], and an increasing number of studies have focused on the crosstalk between glioma progression and the TME. The transmitters, chemokines, and cytokines within the TME could not only enhance tumor progression and invasion, as well as immune evasion but also enhance resistance to therapy [14]. For instance, CCL2 overexpression could reduce TMZ-induced apoptosis by activating AKT signaling, thus leading to TMZ resistance [34]. The activation of the oncogenic pathway in glioma cells could alter cell secretion and thus TME composition. The EGFR pathway plays a key role in the secretion of multiple cytokines and the infiltration of immune cells. EGFR variant III (EGFRvIII) could potentiate IL-1β and IL-6 secretion in GBM cells [35]. For immune cell composition, activation of EGFR signaling could induce CCL2 expression and then elevate the infiltration of tumor-associated macrophages (TAMs) in the TME [12]. It has been found that EGFR-induced changes in the TME can in turn increase the malignant phenotype or drug resistance of glioma cells, which further illustrates the potential therapeutic benefit of targeting EGFR. As a result, it is necessary to have a deeper understanding of the modulation of the EGFR pathway, and the upstream regulation mechanism of EGFR signaling in gliomas remains unclear.

In this study, a comprehensive analysis of RNA-seq data from TCGA and CGGA was conducted to construct an EGFR pathway-related prognostic gene signature, including four genes (MAP3K14, RHOC, STAT1, and VAV3), with a good prediction for clinical outcomes. All the above four genes were reported to be oncogenic in gliomas. Guo et al. discovered that VAV3 can regulate GBM cell proliferation, invasion, and cancer stem-like cell self-renewal [36]. In addition, RHOC was reported to be involved in the downstream of EGFR signaling, and knock-down RHOC can inhibit EGF-induced VEGF expression [37]. Moreover, STAT1 plays a key role in the glioma malignant phenotype, and STAT1 downregulation can inhibit the aggressiveness of GBM cells by regulating the epithelial–mesenchymal transition (EMT) [38]. Furthermore, MAP3K14 can promote cell invasion by regulating mitochondrial dynamics and trafficking [39]. Based on PROGENy (Figure 3G,H), this signature related to four genes can reflect the activation of the EGFR signaling pathway.

There was a positive correlation between the activation of the EGFR-related pathway and the activation of the IFN-γ response pathway. This correlation may be explained by the fact that the downstream of the EGFR and IFN-γ receptors have some overlapping with each other. In hepatocellular carcinoma cells, EGF and IFN-γ both activated PD-L1 expression via the MAPK signaling pathway, which can be blocked by the MEK inhibitor selumetinib [40]. In melanoma, IFN-γ activated the JAK-STAT-IRF1 pathway, resulting in IRF1 binding to PD-L1 promoter [41]. As for EGFR signaling, it triggered the activation of STAT transcription factors [11]. Research has reported that EGF could induce STAT1 expression to exacerbate the IFN-γ-mediated PD-L1 axis in EGFR-positive cancer cell lines, excluding glioma, and blockade of EGFR by afatinib inhibited EGF- and IFN-γ-mediated PD-L1 expression [42]. This downstream overlapping provides a plausible explanation for the positive correlation between the IFN-γ-related and EGFR-related risk scores.

Notably, this study revealed that IFN-γ can indeed upregulate the phosphorylated form of EGFR, indicating that this pro-inflammatory cytokine can serve as an upstream inducer of the EGFR pathway in gliomas. Moreover, the results verified that activation of EGFR can upregulate PD-L1 expression, and thus, the upregulation of EGFR activity may be one of the pathways by which IFN-γ stimulates PD-L1 expression. Although IFN-γ has been reported to lead to increased EGFR activity in other cancer types [21,22], it is notable that this relationship persists in glioma, a CNS tumor with an immune microenvironment completely different from that of peripheral solid tumors. This direct relationship is expected to be one of the therapeutic targets. For example, IFN-γ is one of the indicators that rise in immune cell therapy. Given the dual nature of IFN-γ effects, if its ability to promote PD-L1 expression is inhibited, the efficacy of immune cells can be raised in theory. Based on the findings, inhibition of EGFR may help achieve this goal, which provides a preliminary theoretical basis for the combination of immune cell therapy and EGFR-targeted drugs. A study reported that the blockade of EGFR by afatinib resulted in decreased STAT1 and IRF-1 levels, and disabled the IFN-γ-STAT1-mediated PD-L1 axis in vitro and in vivo for oral cancer and lung cancer [42]. Whether this result can be reproduced in gliomas deserves to be explored in future studies.

In total, 1027 glioma samples were divided into four groups based on EGFR and IFN-γ related signatures. This shows that the combination of two risk scores could predict immune infiltrating cells within the glioma TME. Patients in the high-risk group exhibited low-purity tumors and increased infiltration of immune cells, which indicates that high-risk gliomas induce more immune responses and attract more immune cells to infiltrate due to their greater proliferation and invasion properties. However, it is also important to note that the highest level of inhibitory immune cell infiltration and the highest level of immune checkpoint molecular expression were also observed in the high-risk group. This indicates a strong immunosuppressive TME, which can be reflected by significantly more exhausted T cells in the high-risk group. High activation of these two pathways in the high-risk group may contribute to the high expression of immune checkpoints such as PD-L1. High activation of EGFR may also lead to a more suppressed TME [12,43]. This paradoxical observation suggests that the host has a potentially strong immune response to high-risk gliomas, but it is inhibited by stronger immunosuppressive mechanisms, which highlights the need to reverse the inhibitory TME to unleash the full potential of the intrinsic antitumor immune response. Therefore, from the perspective of immune cell infiltration, it can be speculated that high-risk patients may exhibit a more effective antitumor immune response when receiving immunotherapy, as long as the inhibitory TME is adequately modulated.

The combination of EGFR and IFN-γ related scores is helpful for clinical decision-making. If a patient has a high-risk score, it suggests that this patient may have a poorer prognosis, and a more aggressive treatment should be taken. In addition, as discussed earlier, patients with high-risk scores may be good candidates for immunotherapy when the TME is adjusted. Based on the findings, EGFR-targeted drugs may be a good choice for patients with high-risk scores to have their TME modulated. Thus, patients with high-risk scores may be well suited for a combination of immunotherapy and EGFR-targeted agents, which is currently rarely investigated in gliomas and deserves further exploration.

There are several limitations in this research. First, preliminary in vitro experiments were only conducted to validate the activation of EGFR by IFN-γ. The specific mechanism by which IFN-gamma leads to the elevation of phosphorylated EGFR was not explored, which is important for elucidating the clear interaction between the two and deserves further investigation in vitro and in vivo in future studies. Second, the expression of immune checkpoint molecules and immune cell content in the TME was calculated by RNA expression data. Future studies could validate the conclusions at the protein level using patient specimens and immunostaining techniques.

In conclusion, this study provides novel insights into the relationship between IFN-γ-related and EGFR-related pathways in glioma patients. Moreover, the results demonstrate that IFN-γ could be an upstream inducer of EGFR signaling activation. Based on this relationship, EGFR-related and IFN-γ-related signatures were jointly used to divide the glioma patients. High-risk patients tended to have poorer prognosis and more inhibitory TME. Therefore, these patients may be more suitable for immune checkpoint blockade (ICB) therapy or other immunotherapeutic approaches, which warrants further validation for a clinical cohort in a follow-up study.

## Data Availability

All data and materials used are available from the corresponding author upon reasonable request.

## Supplementary Materials

The following supporting information can be downloaded at: https://www.mdpi.com/article/10.3390/brainsci13091349/s1, Supplementary Figure S1. The process of constructing EGFR-related signature. Supplementary Figure S2. The process of constructing IFN-γ response-related signature.
