# Supplementary figures and images for: The Crosstalk between the EGFR and IFN-γ Pathways and Synergistic Roles in Survival Prediction and Immune Escape in Gliomas
Source: Brain Sci. 2023 Sep 20;13(9):1349. doi: 10.3390/brainsci13091349 (PMC10526459; doi:10.3390/brainsci13091349)

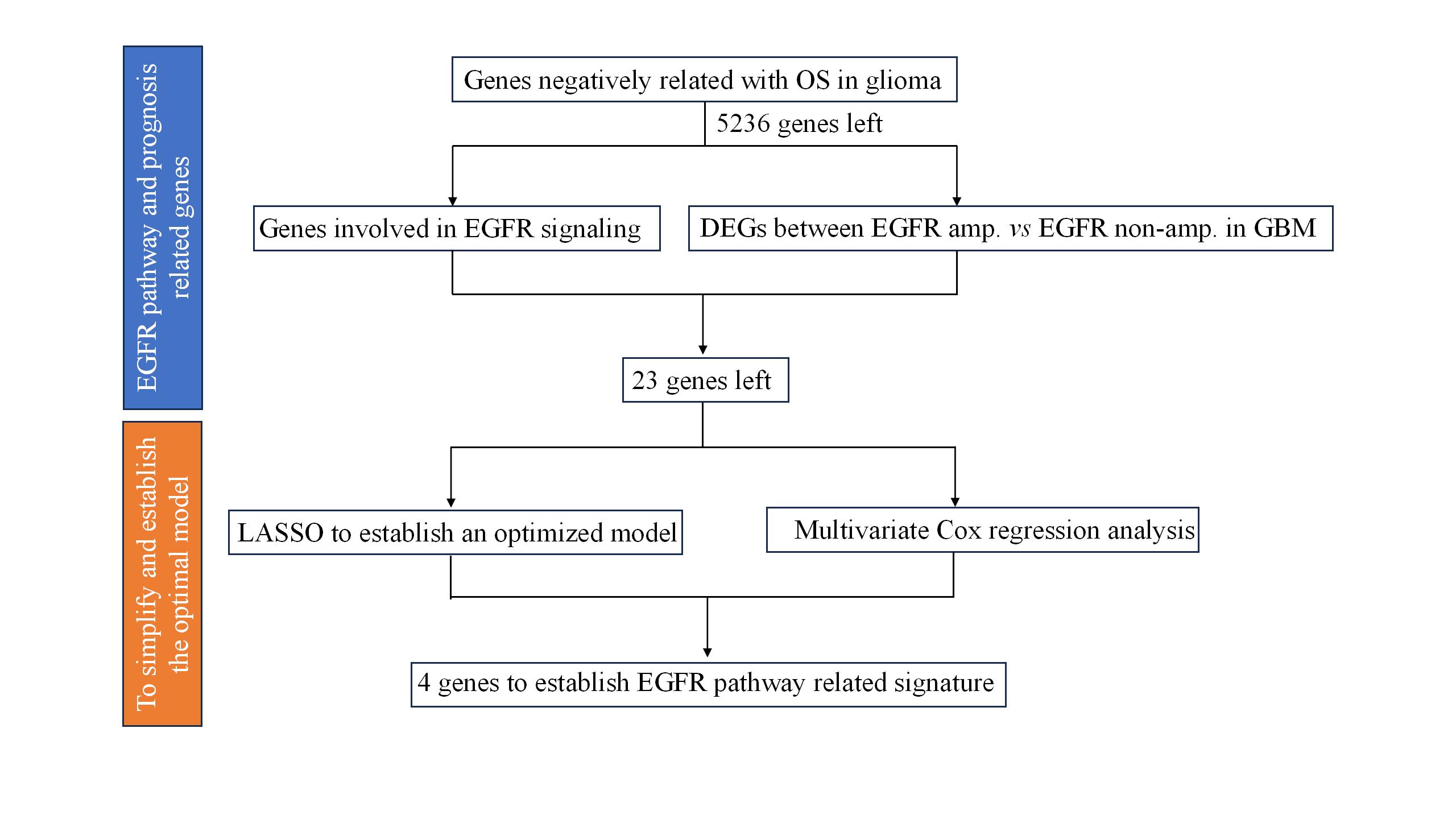

Supplement: Supplementary file 1 [file brainsci-13-01349-s001.zip › Supplemental Figure S1.jpg]

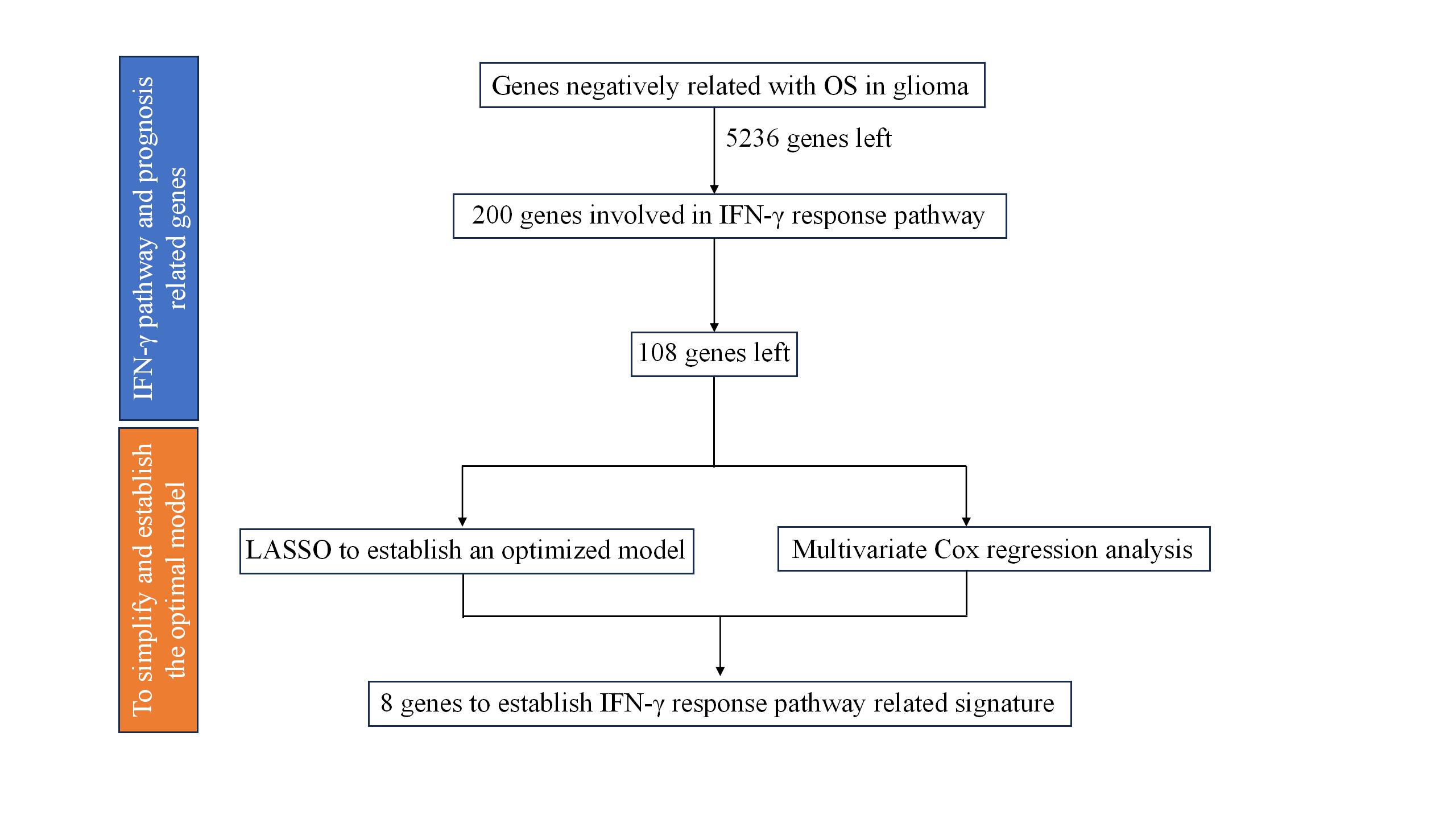

Supplement: Supplementary file 1 [file brainsci-13-01349-s001.zip › Supplemental Figure S2.jpg]
